# University of Pennsylvania

**Published:** 1843-05-13

**Authors:** Daniel C. Holliday


### CLINICAL LECTURES AND REPORTS.

### UNIVERSITY OF PENNSYLVANIA.

#### CLINIC OF DR. W. POYNTELL JOHNSTON.

*At the Locust Street Dispensary, April* 12, 1843.

[Reported by Daniel C. Holliday.]

Case I.—*Varicose Veins of the Left Leg.*

Bernard Camppe, aged 35, strong constitution, by trade a carter, and accustomed to use the left leg in digging, has perceived for a number of years a gradual increase in the volume of the left saphena vein; about the same period he experienced occasional pains along the course of the right sciatic nerve, which gradually occurred more frequently, and were of longer duration; while, at the same time, he noticed a swelling in each iliac region, which he attributed to the constant jolting of the cart, and the weight of the bowels.

Upon examination at the clinic, the left saphena and all its branches were found in an extremely varicose condition, from the internal malleolus to its junction with the femoral vein; below the internal condyle the vein offered innumerable convolutions, several of which were collected into tumors of the size of a small egg. Above the condyle the vein was tortuous, very much enlarged, and when grasped between the fingers, offered almost the resistance of cartilage, so much were the coats thickened; at the point of junction with the femoral the saphena appeared constricted, but above this point the femoral itself could be traced in a similar varicose condition, under Poupart's ligament, into the iliac fossa.

On each side of the rectus abdominis, above Poupart's ligament, appeared a projecting tumor, caused, it would seem, by the gradual distension of the combined tendons of the abdominal muscles at these points. When the patient assumed the erect position the abdominal viscera apparently fell forwards into these pouches, which disappeared when the patient was placed upon his back; in the recumbent position no tumor could be felt in the iliac regions, and the varicose appearance of the saphena was removed.

On the right side the patient complained of pain in the gluteal sciatic nerves, and he was able to trace with precision the whole course of this nerve, as giving him pain and causing numbness of the right foot. This pain was materially increased by coughing, or any effort producing mechanical compression of the lower part of the abdomen.

The saphena vein is not unfrequently rendered varicose in athletic individuals, by the constant state of tension in which the muscles of the leg are kept, from the nature of the occupations of such persons; two causes co-operate to produce this condition: 1st, a greater amount of blood than usual is attracted to the leg and foot by the constant exercise of their muscles; and 2d, the whole, or nearly the whole of this blood is required to be returned to the heart, against the resistance of gravitation by the saphena alone, because the satellite veins being compressed by the contracted muscles, are unable to perform their part. Under these circumstances the saphena dilates, its coats become thickened, and a permanent varicose condition is established.

This explanation, however, would not suffice in the case presented, although the occupation of the individual is such as might favour it. He is employed in digging cellars, and while digging uses the left leg; but the varicose condition extends throughout the whole course of the saphena vein, and beyond this along the femoral vein into the iliac region. Now it is obvious that the cause of obstruction to the circulation in the veins of the left lower extremity must be sought at some point between the commencement of the dilation and the heart. It cannot be supposed to exist in the vena cava itself, because the veins of the opposite extremity are unaffected; it should, therefore, be found between the cava and the femoral, that is to say, in the iliac fossa. The tumor formed in this region by the imperfectly supported viscera, offers a probable cause of compression of the left iliac vein, which would abundantly account for the symptoms exhibited.

On the right side a similar tumor exists, and yet the vein has escaped; it has escaped, however, at the expense of the ischiatic plexus of nerves, causing cramps and pain along the course of the gluteal and sciatic nerves of this side.

The various methods of treatment for this condition of the veins, which is apt to produce and maintain an indolent ulcer of the leg, were described.

They consist of the ligature, the section, the excision and the compression of the vein.

The ligature of a vein is on high authority almost proscribed; still, this proscription must not be rendered too absolute; a healthy vein may frequently be tied with impunity, while the ligature of a varicose vein, the coats of which are thickened and diseased, is too frequently followed by death. The same is true of all the operations which interest the lining membrane of a diseased vein. All these operations have a common object, the obliteration of the main trunk of the vein through the medium of a local inflammation. They all offer a common danger, that this local inflammation may generalise itself, and death ensue from a purulent phlebitis.

Independently of the danger to life of such operations, there is another objection, viz.; that they frequently fail of their object; 1st, because the obliteration of one or more trunks may only produce a similar varicose condition of those which remain, the original cause continuing. And 2d, because the obliteration itself is not always effected by the operation. Thus the operation of Brodie is frequently unsuccessful, because the two severed extremities of the vein may reunite, and even the ligature itself is not free from such a result.

In 1833 a man was admitted into the Hospital of St. Louis, in the service of Prof. Gerdy, for a varicose ulcer of the leg. Ten years before, for a similar ulcer, the saphena had been tied by M. Berard opposite the condyle. The account of the operation by the patient was clear; the application of the ligature—the day on which it came away—the effects upon the ulcer, which was cured, and remained so for seven years—were all accurately described; and to confirm the accuracy of his description, the vein was found constricted opposite the condyle; a cicatrix existed in this point, and yet the permeability of the vein was reëstablished, as could easily be recognised by placing a finger upon the vein above and below the strictured point, and causing the blood to flow backwards and forwards between the two.

The operation of Davat is less objectionable, as being less dangerous than those described, yet it is not devoid of danger; the first results of this operation were extremely flattering,—and although subsequent experience has shown that it is occasionally followed by fatal results, still the proportion of successful cases is so great that we are induced to look for some cause to explain the difference in result between this operation and those in which the lining membrane of the vein is directly interested. If a pin be placed beneath the vein and a twisted suture above it, the sides of the vein are brought in contact without lesion of its coats. Is it possible that under these circumstances the vein is obliterated without the aid of inflammation? that the complete arrest of circulation causes coagulation of the blood, and that the coagulum undergoes the same changes as would follow an entire arrest of circulation in an artery, viz., the absorption of the fluid particles and the organisation of the fibrin? That is to say, may the vein be converted thus into a solid cylinder and cease to be permeable, without the production of inflammation, and, consequently, without danger of a general phlebitis?

In the case presented no operation can be performed, because the point of obstruction is in the iliac fossa, and beyond our reach. We must, therefore, be content to give to the varicose veins an artificial support, to compensate for that which they have lost by the gradual yielding of their own parietes. The limb was elevated to evacuate the distended vein. A bandage was applied from the foot to the groin, and then made to encircle the lower portion of the abdomen, so as to sustain the abdominal viscera. Cups were directed to the spine on the right side, and along the course of the painful gluteal and sciatic nerves; the bowels to be kept free with the comp. cathartic pill. Should the compression prove of essential benefit, a more permanent and efficient apparatus than a mere roller would be applied.

CASE II.—*Mucous Polypus of the Nose, removed by the Forceps.*

The patient, —— Robinson, ætat. 23, in the enjoyment of excellent health, was attacked six weeks since with a cold in the head. From this period he stated that he had not been able to breathe through the left nostril, and had lost the sense of smell on the same side. He was sensible of an obstruction on the left side, which he endeavoured ineffectually to remove by continual efforts. During these efforts there could be perceived in the left nostril, by examination in a strong light, a pellucid, bladder-like mass, which moved backwards and forwards during inspiration and expiration. The volume of this tumor increased in moist weather; it was unaccompanied by pain or hemorrhage; and even when rudely touched with the finger or a probe did not bleed.

This was clearly a case of polypus of the nose, but polypi differ in character. The mucous membranes are liable to several varieties, and of each of these the Schneiderian membrane presents examples. There occasionally arises in the nostril a fleshy growth of a red colour, extremely painful, and bleeding spontaneously, or upon the slightest touch, constituting the fleshy polypus of authors. This species of polypus grows rapidly, and is highly organised, as is exhibited by its vascularity and the pain which is inherent to the tumor itself, and is not dependent on pressure upon the surrounding parts. This species is very apt to degenerate into cancer, and is probably a malignant growth from the commencement. From this form of polypus the case presented differed in every feature.

There is another species of hard polypus to which all mucous membranes are liable, but which is found most frequently in the womb, and occasionally in the nostrils or pharynx. It is fibrous in texture; not vascular; free from pain, except such as is produced by pressure upon the parietes of the cavity in which it is contained; susceptible of carcinomatous degeneration, although this is not of frequent occurrence; and when presented, generally occupies that portion which is most distant from the pedicle or base by which the polypus is attached, and does not occupy the whole tumour. When this degeneration exists the polypus may be accompanied by hemorrhage. With this variety, again, the case under examination could not be confounded.

There remained but one more species of polypus, viz., the soft polypus. With this variety, in its incipient stage, the case of Robinson corresponded entirely. The soft or mucous polypus presents the aspect, but for its colour, of a mere hypertrophy of the mucous membrane. When examined, however, by dissection, it is found to be devoid of bloodvessels, and to offer no trace of organisation. When removed, it resembles very accurately in appearance an oyster. The difference in the mode of growth of these different varieties of polypi, with their effects upon the parietes of the cavities in which they originated, were described, and the different methods of treatment commented upon. The case presented offered no peculiarity, except probably the rapidity of its growth, and corresponded accurately with the description of mucous polypus given by authors.

The polypus was then removed with the forceps; after which, by means of the instrument of Bellocque, a large pledget of lint was introduced into the posterior nares, and made forcibly to sweep the nostril from behind forwards, to remove any trace of the disease which might have escaped the action of the forceps.

In order to confirm the cure, and prevent a return of the disease, the patient was directed to use the following snuff.

℞. Pulv. Nicotian. Tabac., ℥ss.; Cupri Sulphatis, ʒj. M. S. Use as a snuff.

